# Incidence and Risk Factors of Post-endoscopic Retrograde Cholangiopancreatography (ERCP) Acute Pancreatitis in a Mexican Secondary-Level Hospital: A Retrospective Study

**DOI:** 10.7759/cureus.97736

**Published:** 2025-11-25

**Authors:** Julio C Balderas-Ortega, Victor M Ayuso-Diaz, Jose A Gameros-Martinez., Kenneth Aleman-Paredes

**Affiliations:** 1 Department of Surgery, IMSS (Instituto Mexicano del Seguro Social) Regional General Hospital No. 220 “Gral. José Vicente Villada”, Toluca, MEX; 2 Research and Education Division, Medical Care and Research, Mérida, MEX; 3 Genomic-Metabolic Unit, Universidad Marista de Mérida, Mérida, MEX

**Keywords:** difficult cannulation, endoscopic retrograde cholangiopancreatography, ercp complications, post-ercp pancreatitis, precut sphincterotomy, risk factors

## Abstract

Introduction: This study aimed to determine the incidence and factors associated with acute pancreatitis following endoscopic retrograde cholangiopancreatography (ERCP) in a second-level hospital in Mexico, where local data are limited compared to tertiary centres.

Methods: A single-centre retrospective study was conducted, including 52 patients with a native papilla who underwent ERCP between January and August 2024. Patients with previous ERCP, altered anatomy, or active biliary pancreatitis were excluded. Clinical and procedural variables were collected, and post-ERCP pancreatitis (PEP) was defined according to the Cotton consensus criteria (abdominal pain with amylase ≥3× upper limit of normal). Univariate and multivariate logistic regression analyses were performed to identify associated risk factors. The cumulative effect of multiple risk factors on PEP incidence was also explored. The short study period and small sample size were acknowledged as limitations that may affect generalisability.

Results: Four cases of PEP were identified (7.7%), all classified as mild. Univariate analysis revealed significant associations with precut use (p=0.012), rectal indomethacin administration (p=0.001), and more than three cannulation attempts (p<0.001). In multivariate analysis, only >3 cannulation attempts remained statistically associated with increased PEP risk (OR 6.2; 95%CI: 1.2-33.3; p=0.032). The incidence of PEP increased progressively with the number of risk factors present (0% for none, 3.2% for one, 12.5% for two, and 25% for three), although this trend was not statistically significant due to the limited number of events. All affected patients recovered fully after conservative management, without intensive care or surgical intervention.

Conclusions: The observed incidence of PEP was consistent with that reported in international series. Cannulation attempts exceeding three were statistically associated with an increased risk of PEP. The apparent association of rectal indomethacin with PEP likely reflects confounding by indication, as its use was concentrated in high-risk patients. These findings highlight the need for multicentre studies to confirm these associations and reinforce the implementation of standardised prophylactic strategies, even in secondary-level hospitals.

## Introduction

Endoscopic retrograde cholangiopancreatography (ERCP), introduced in the 1970s, has evolved into a highly specialised endoscopic procedure [1]. It is currently the preferred technique for the minimally invasive management of various pancreatobiliary diseases, including choledocholithiasis, cholangitis, benign and malignant biliary strictures, and functional disorders of the sphincter of Oddi [2,3].

Despite its therapeutic efficacy and favourable cost-benefit profile compared to surgical alternatives, ERCP is associated with a significant risk of adverse events. The most frequent and clinically relevant complication is post-ERCP pancreatitis (PEP) [4]. The incidence of PEP ranges from 3% to 10% in most international studies and can exceed 20% in high-risk populations, such as young women and patients with sphincter of Oddi dysfunction [5]. This variability reflects differences in diagnostic criteria, procedural techniques, operator experience, and patient characteristics [6, 7].

Several clinical and technical factors have been associated with an increased risk of PEP. Clinical factors include female sex, age under 60 years, and a history of pancreatitis. Technical factors include difficult cannulation, inadvertent pancreatic duct injection, the precut sphincterotomy technique, absence of pharmacological prophylaxis with non-steroidal antiinflammatory drugs (NSAIDs) such as indomethacin or diclofenac, and omission of prophylactic pancreatic stent placement [6,8,9].

In response, international societies such as the European Society of Gastrointestinal Endoscopy (ESGE) and the American Society for Gastrointestinal Endoscopy (ASGE) have recommended preventive strategies based on pharmacological and technical measures, including rectal administration of NSAIDs and prophylactic pancreatic stenting [5,7].

In Mexico, available evidence on the incidence and risk factors of PEP is limited and primarily derived from tertiary-level hospitals. For instance, González-González et al. reported complication rates comparable to those observed in high-specialty centres internationally, although variability in access to specialised resources may influence outcomes [10].

In contrast, second-level hospitals, defined in the Mexican healthcare system as regional institutions offering intermediate complexity care with limited access to advanced endoscopic resources, remain underrepresented in the literature. Differences in infrastructure, procedural volume, clinical protocols, and operator training may result in divergent epidemiological profiles compared to referral centres [3,4].

Moreover, the systematic implementation of prophylactic strategies recommended by international guidelines remains inconsistent in many Mexican hospitals. Limited availability of rectal NSAIDs, lack of established institutional protocols, and low awareness among healthcare providers have been documented as barriers to adherence. These trends have been reported in regional surveys and narrative reviews in Latin America [4,8].

Therefore, local data from second-level hospitals are crucial to determine the actual incidence of PEP and the factors that contribute to its development in these settings. This knowledge is essential to contextualise clinical practice, optimise the use of limited resources, and guide the implementation of effective prophylactic measures. This study aims to determine the incidence of PEP and to identify associated risk factors in patients undergoing ERCP at a second-level hospital in Mexico.

## Materials and methods

This was a single-centre retrospective study that included patients with native papilla who underwent ERCP at the Hospital General Regional No. 220 “General José Vicente Villada” of the Instituto Mexicano del Seguro Social (IMSS), Toluca, Mexico, between January and August 2024. All procedures were performed on an elective basis. 

Study population

The inclusion criteria were patients with native papilla who underwent ERCP at the Hospital General Regional No. 220 “General José Vicente Villada” of the Instituto Mexicano del Seguro Social (IMSS) during the study period. Exclusion criteria were as follows: (i) patients with a history of previous ERCP, (ii) patients with Billroth II or Roux-en-Y surgical reconstruction, (iii) patients with active biliary pancreatitis at the time of the procedure, and (iv) patients in whom cannulation was failed or incomplete. These cases were excluded because, in our institution, ERCPs without endoscopic findings are often performed under incomplete diagnostic evaluation or with significant missing procedural data, limiting accurate assessment of cannulation difficulty, papillary trauma, and pharmacological prophylaxis. Including such cases could introduce misclassification bias; therefore, only procedures with complete clinical and endoscopic documentation were retained for analysis.

Only those patients with complete clinical records, documented follow-up, and availability of endoscopic reports for retrospective analysis were included. Missing data were cross-checked with hospital records, and any unclear entries were resolved by consensus among two independent investigators. All eligible cases were included to minimise selection bias. This resulted in a total of 52 patients included in the study.

Endoscopic procedure

All ERCP procedures were performed by board-certified gastroenterologists with at least five years of experience in therapeutic endoscopy, assisted by specialised nursing staff from the Endoscopy Department of Hospital General Regional No. 220 (IMSS). Standardised techniques for cannulation of the biliary tract were employed, including sphincterotomy and precut sphincterotomy when conventional cannulation was unsuccessful. Depending on the clinical and technical characteristics of the procedure, rectal indomethacin (100 mg) was administered prophylactically. In selected cases, placement of a pancreatic stent was indicated and recorded.

Data collection

Data sources included endoscopic reports and institutional clinical records. To improve data reliability, all variables were reviewed by two independent investigators. Ambiguous or incomplete entries were clarified through consensus or excluded. The number of cannulation attempts was defined according to ESGE guidelines, considering each passage of the guidewire through the papilla as one attempt.

Study definitions

Post-ERCP Pancreatitis

The diagnosis and severity grading of PEP were established according to the consensus criteria of Cotton et al. [11]. PEP was defined as new or worsened abdominal pain persisting for at least 24 hours after the procedure, associated with serum amylase levels ≥3 times the upper limit of normal, measured 24 hours post ERCP. Severity was categorised as mild (≤3 days of hospitalisation), moderate (4-10 days), or severe (>10 days).

Sphincterotomy With Precut

This was defined as needle-knife access to the biliary tract in the papillary region after ≥5 unsuccessful cannulation attempts, based on operator discretion. For standardisation, the actual number of attempts prior to precut was recorded, and the mean number across all patients undergoing this technique was calculated.

Statistical analysis

Univariate analysis was performed using the chi-square test or Fisher’s exact test for categorical variables and Student’s t-test for continuous variables. Variables with p < 0.05 in univariate analysis and those identified as established risk factors for PEP in the ESGE guidelines were included in a multivariate logistic regression model to identify independent predictors. Multicollinearity was assessed using variance inflation factors, and cases with missing data for key variables were excluded from the final model. The threshold for statistical significance was set at p < 0.05 (two-tailed). Analyses were conducted using IBM SPSS Statistics for Windows, version 28.0 (Released 2021; IBM Corp., Armonk, New York, United States).

## Results

Indications for ERCP

A total of 52 patients were included. The main indications for ERCP were symptomatic choledocholithiasis in 30 patients (57.7%), acute cholangitis in 11 (21.2%), defined according to the Tokyo Guidelines 2018 [12], obstructive jaundice without cholangitis in eight (15.4%), and recurrent pancreatitis with suspected sphincter of Oddi dysfunction in three (5.8%), diagnosed based on clinical suspicion (manometry was not performed). The distribution of ERCP indications did not significantly differ between patients with and without PEP.

Incidence and severity of PEP

PEP occurred in four out of 52 patients (7.7%; 95%CI: 2.1-18.5%). All cases were classified as mild, with no requirement for intensive care or surgical intervention. Hospital stays were short and managed conservatively, with all patients achieving full recovery without complications.

Risk factors associated with PEP

Univariate Analysis

Univariate analysis was conducted to identify clinical and technical variables associated with the occurrence of PEP (Table 1). Precutting was performed in nine patients overall, of whom three developed PEP (3/4, 75%) and the remaining six were without PEP (6/48, 12.5%) (p = 0.012). Indomethacin was administered in four patients, with two developing PEP in each group (2/4, 50% vs. 2/48, 4.1%; p = 0.001). The paradoxical association between indomethacin and increased risk likely reflects confounding by indication, whereby high-risk patients preferentially received NSAIDs. Cannulation attempts exceeded three in 14 patients; all four cases of PEP occurred in this subgroup (4/4, 100%), and 10 were from the group without PEP (10/48, 20.8%) (p < 0.001). Interpretation of these associations should be made with caution, given the small sample size and limited statistical power.

**Table 1:** Univariate analysis of clinical and technical characteristics in patients with and without PEP. Univariate analysis explored the association between clinical-demographic and procedural factors and the development of PEP. Patients who developed PEP were compared to those who did not. The analysed variables included age, sex, comorbidities (type 2 diabetes mellitus, systemic arterial hypertension, and obesity), as well as procedural characteristics such as pre-cut sphincterotomy, number of cannulation attempts, biliary stent placement, and the use of prophylactic NSAIDs. P-values were calculated using the Student’s t-test for continuous variables (expressed as mean ± SD) and the chi-square test for categorical variables. Statistical significance was defined as p < 0.05. PEP: post-ERCP pancreatitis; ERCP: endoscopic retrograde cholangiopancreatography; NSAIDs: non-steroidal anti-inflammatory drugs

Risk factor	Without PEP (n=48)	With PEP (n=4)	p-value	Test Statistic (χ² / t)	Statistical Test
Precut during ERCP	6 (12.5%)	3 (75%)	0.012	χ² = 4.61	Chi-square
Use of indomethacin	2 (4.1%)	2 (50%)	0.001	χ² = 10.23	Chi-square
Cannulation attempts >3	10 (20.8%)	4 (100%)	<0.001	χ² = 12.86	Chi-square
Age, mean ± SD (years)	59.1 ± 12.3	51.7 ± 9.4	0.482	t = 0.71	Student’s t-test
Female sex	26 (54.1%)	3 (75%)	0.628	χ² = 0.24	Chi-square
Arterial hypertension	7 (14.5%)	1 (25%)	0.484	χ² = 0.49	Chi-square
Type 2 diabetes mellitus	5 (10.4%)	1 (25%)	0.381	χ² = 0.77	Chi-square
Obesity	3 (6.2%)	0 (0%)	0.638	χ² = 0.22	Chi-square
Biliary stent placement	7 (14.5%)	1 (25%)	0.484	χ² = 0.49	Chi-square
Use of diclofenac	5 (10.4%)	0 (0%)	0.487	χ² = 0.48	Chi-square

There were no significant differences in age, sex, arterial hypertension, type 2 diabetes mellitus, obesity, biliary prosthesis placement, or diclofenac use between the PEP and non-PEP groups.

Multivariate Analysis

The three variables found to be significant in the univariate analysis (precutting, indomethacin use, and >3 cannulation attempts) were included in a multivariate logistic regression model (Table 2). Among these, only the number of cannulation attempts remained statistically associated with the development of PEP (OR 6.2, 95%CI: 1.2-33.3; p = 0.032). However, the wide confidence interval reflects uncertainty due to the small sample size. Precutting (OR 2.8, 95%CI: 0.7-11.2) and indomethacin use (OR 1.5, 95%CI: 0.3-7.5) did not retain statistical significance after adjustment. Key variables identified in high-quality evidence (e.g., American Gastroenterological Association (AGA) meta-analysis, ASGE guidelines), such as guidewire passage into the pancreatic duct, contrast injection, or cannulation time, were not available in this retrospective cohort and thus represent a limitation.

**Table 2:** Multivariate analysis of factors associated with post-ERCP pancreatitis. This table shows the results of the logistic regression model, including the variables that were significant in the univariate analysis. The model revealed that more than three cannulation attempts are an independent risk factor for the development of post-ERCP pancreatitis. Although relevant in the univariate analysis, the variables precut and indomethacin did not retain statistical significance after multivariate adjustment. ERCP: endoscopic retrograde cholangiopancreatography

Variable	OR	95% CI	p-value
Cannulation attempts >3	6.2	1.2–33.3	0.032
Precut during ERCP	2.8	0.7–11.2	ns
Use of indomethacin	1.5	0.3–7.5	ns

Cumulative effect of risk factors

**Figure 1:**
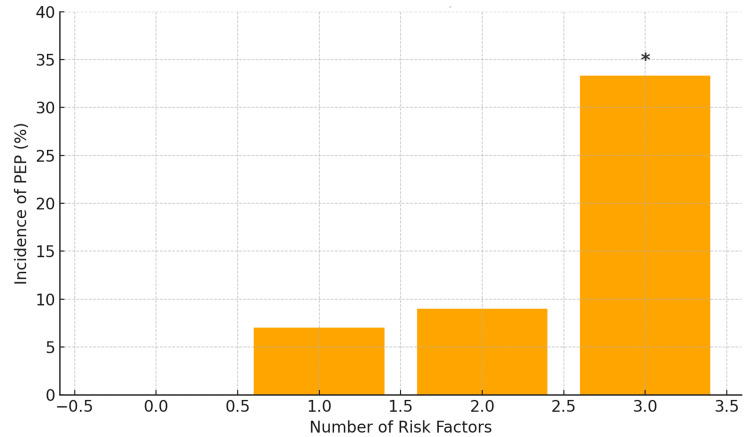
Incidence of post-ERCP pancreatitis according to the number of risk factors present. Proportion of cases of PEP observed according to the number of clinical and technical risk factors present in each patient. This visualization allows dimensioning how the frequency of complications varies in relation to the accumulation of associated elements, without implying a causal relationship or statistical significance. *p < 0.05, Chi-square test comparing incidence across groups PEP: post-ERCP pancreatitis; ERCP: endoscopic retrograde cholangiopancreatography

When analysed cumulatively, the incidence of PEP increased with the number of risk factors present (Figure 1). Among patients with no risk factors (n = 31), none developed PEP (0%, 95%CI: 0-11.2%). Among those with one factor (n = 10), one developed PEP (10%, 95% CI: 0.3-44.5%); with two factors (n = 8), one developed PEP (12.5%, 95% CI: 0.3-52.7%); and with all three factors (n = 3), two developed PEP (66.7%, 95% CI: 9.4-99.2%). While this trend suggests a potential additive effect, it should be considered exploratory rather than confirmatory due to the small number of events.

## Discussion

The incidence of PEP seen in this study is within the expected range according to the international literature, which reports rates between 3% and 10% in the general population. It is also consistent with multicentre studies, such as that of Cebi et al., in which the incidence of ERCP-related complications remained within these margins in European centres [2]. Notably, all reported cases were classified as mild and did not require intensive therapy or surgical intervention, consistent with previous studies indicating a self-limiting clinical course in most cases of PEP [4]. However, given the relatively small sample and low number of events result in wide confidence intervals, it should be noted that this estimate has a wide margin of uncertainty and cannot exclude relevant deviations from the true incidence in similar secondary-level settings [7].

Regarding risk factors, the univariate analysis revealed a significant association between PEP and precut technique, rectal indomethacin administration, and more than three cannulation attempts. However, in the multivariate model, only more than three cannulation attempts remained as an independent predictor (OR: 6.2; 95%CI: 1.2-33.3; p = 0.032). This finding is consistent with reports by Tryliskyy et al., who demonstrated that cannulation difficulty is a major contributor to PEP risk, likely due to increased papillary trauma and pancreatic duct exposure [8]. Similarly, Akshintala et al. showed that the risk of PEP increases progressively with each failed cannulation attempt, particularly when three or more attempts are exceeded [9]. It is worth noting that the wide confidence interval reflects substantial statistical uncertainty, and this association should be confirmed in larger studies. These findings reinforce the importance of procedural standardisation and the need to adopt strict limits on cannulation attempts, especially in secondary-level hospitals.

The role of precutting in PEP remains controversial in the literature. In our study, it reached statistical significance in the univariate analysis but lost significance in the multivariate model. This could reflect an indirect association driven by overall procedural complexity, rather than by the precut technique itself. In line with our findings, studies such as those by Sanders et al. [4] and Lorgulescu et al. [13] suggest that early precutting, when performed by experienced endoscopists, does not necessarily increase the risk of PEP. While operator experience was not homogeneously recorded in our sample, it remains a potential confounder influencing outcomes in precut procedures.

Rectal indomethacin, although widely endorsed by international societies such as ESGE [14] and ASGE [15,16] as a cost-effective prophylactic measure, was paradoxically associated with PEP in our univariate analysis. This likely reflects an indication bias, as its use was concentrated in high-risk patients. Similar observations have been reported in Latin American cohorts, such as the study by Tlatoa-Ramírez et al., which also suggested that NSAID administration tends to be reserved for patients with multiple risk factors, thereby distorting its apparent protective effect in non-adjusted analyses [17]. The small number of patients who received indomethacin in our study further limits the strength of this association and should be interpreted with caution.

An upward trend in PEP incidence was observed with the accumulation of clinical and technical risk factors, reaching 25% in patients with three or more. Although this trend was not statistically significant, likely due to limited statistical power, it supports the hypothesis of an additive effect, as described in previous literature [18,19]. This cumulative risk approach aligns with prediction models proposed by authors such as Thiruvengadam et al. [19] and reinforces the need to identify high-risk profiles pre-procedure.

Most Mexican studies to date have focused on tertiary-level institutions. For instance, the work of González-González et al. at a national referral centre reported similar PEP incidences and emphasised the relevance of standardising endoscopic techniques and prophylactic measures in high-complexity settings [10]. However, the reality in second-level hospitals remains largely unexplored. Our study contributes to bridging this gap by providing context-specific data, highlighting unique challenges in settings with limited access to specialised technology or trained personnel. Furthermore, reports by Nicolás-Pérez et al. [20] and Peláez-Luna et al. [21] have underscored the low adherence to PEP prevention protocols in Mexico. This includes suboptimal use of NSAIDs and prophylactic pancreatic stents, attributable to the absence of institutional guidelines, budgetary constraints, and limited awareness of current recommendations among healthcare providers.

Finally, several limitations must be acknowledged. The retrospective design, small sample size, and low event rate reduce the power of the study to detect subtle associations and result in wide confidence intervals. Additionally, we could not consistently document operator experience, which may have influenced key outcomes such as success in cannulation or safety of precut techniques. Despite these limitations, this study offers novel insights from a second-level hospital and provides a foundation for future multicentre prospective studies tailored to the Mexican healthcare context.

## Conclusions

This retrospective study, conducted at a second-level hospital within the Mexican healthcare system, observed an incidence of PEP of 7.7%. All cases were classified as mild, indicating that although PEP remains a significant complication, it is self-limiting when identified and managed promptly. The analysis identified more than three cannulation attempts as the only independent risk factor, emphasising the importance of optimising endoscopic technique and limiting prolonged papillary manipulation. While the use of precutting and rectal indomethacin administration was associated with PEP in the univariate analysis, this association was not evident following multivariate adjustment. This suggests that these factors may indicate technically complex procedures rather than being direct causal factors. Finally, the increasing trend in PEP incidence according to the cumulative number of risk factors present underscores the need for individualised risk assessment prior to the procedure and the judicious use of preventive measures. Although these findings are limited by sample size, they provide valuable local evidence and highlight areas for improvement in endoscopic practice in settings with limited resources.
